# A Case of Avian Influenza Co-Infection and Multifactorial Diseases in a Broiler Chicken Farm in Majalengka, West Java, Indonesia

**DOI:** 10.3390/vetsci13040364

**Published:** 2026-04-08

**Authors:** Tyagita Hartady, Sarah Darmawan Sugandi, Muhammad Viqih

**Affiliations:** 1 Department of Biomedical Science, Faculty of Medicine, Universitas Padjadjaran, Jatinangor, Sumedang 45363, Indonesia; 2 Study Program of Veterinary Medicine, Faculty of Medicine, Universitas Padjadjaran, Jatinangor, Sumedang 45363, Indonesia; sarah21005@mail.unpad.ac.id; 3 PT. New Hope Farm Indonesia, Pelayangan, Gebang, Cirebon 45191, Indonesia; drh.viqih@gmail.com

**Keywords:** avian influenza, broiler chickens, co-infection, multifactorial disease, serology, environmental stress

## Abstract

Poultry disease outbreaks are frequently multifactorial, involving complex interactions between infectious agents and environmental stressors. This report describes a case of avian influenza (AI) co-infection in a commercial broiler farm located in Majalengka, West Java, Indonesia, where a total of 11,000 birds exhibited increased mortality during the grow-out period. Diagnostic investigations included pathological examination, serological testing—such as hemagglutination inhibition (HI) assays for AI H5 and H9, enzyme-linked immunosorbent assay (ELISA) for infectious bronchitis, and rapid testing for Mycoplasma gallisepticum—Polymerase Chain Reaction (PCR) analysis, fecal flotation for coccidiosis, and an environmental assessment measuring ammonia levels, humidity, and heat stress index. Pathological findings revealed tracheitis, airsacculitis, thymitis, bursitis, perihepatitis, ascites, petechial hemorrhages, nephromegaly, and enteritis, indicating a complex disease process. Serological results demonstrated exposure to AI H9 and H5, with differing infection dynamics, while the uneven distribution of infectious bronchitis antibodies suggested field challenges. Additionally, partial exposure to Mycoplasma gallisepticum was observed. PCR results were negative at the time of sampling, consistent with post-infection phases. Environmental evaluation revealed elevated ammonia levels, excessive humidity, and a high heat stress index. In conclusion, the increased mortality was associated with avian influenza co-infection, complicated by secondary infections and adverse environmental conditions.

## Simple Summary

This study reports a disease outbreak in a commercial broiler chicken farm in Majalengka, West Java, Indonesia, characterized by increased mortality during the growing period. The objective was to investigate the causes of the outbreak and to understand how multiple factors contributed to disease severity. Examination of affected chickens revealed lesions in the respiratory system, immune organs, kidneys, and digestive tract, suggesting a complex disease process rather than a single infection. Laboratory analyses indicated that the flock had been exposed to various avian influenza viruses and infectious bronchitis virus at different times, while some birds were concurrently affected by bacterial respiratory infections. Environmental assessment revealed poor air quality, high humidity, and heat stress inside the poultry house—conditions known to compromise the natural defenses of chickens. Overall, this case demonstrates that disease outbreaks in broiler production are often driven by interactions between infectious agents and unfavorable environmental conditions. These findings highlight the importance of integrated disease control strategies, including proper housing management, environmental monitoring, and early health surveillance, to minimize economic losses and improve poultry welfare.

## 1. Introduction

Poultry production is integral to food security, as it provides affordable and high-quality animal protein through meat and eggs [1]. The productivity of broiler chickens is heavily influenced by various factors, including nutrition, water quality, housing management, biosecurity measures, and overall health status. Disruptions in any of these elements can result in reduced performance, elevated mortality rates, and significant economic losses within the poultry industry [2]. Infectious diseases such as Avian Influenza (AI), Infectious Bronchitis (IB), Chronic Respiratory Disease (CRD), colibacillosis, and coccidiosis pose major health challenges to broiler production globally, particularly in regions with intensive farming systems [3]. These challenges are often worsened by poor environmental conditions, which serve as critical predisposing factors.

Avian Influenza is a highly significant viral disease affecting poultry, caused by Influenza A viruses from the family Orthomyxoviridae, exhibiting a broad spectrum of pathogenicity [4]. Since 2004, Indonesia has experienced endemic circulation of highly pathogenic AI H5N1, while low pathogenic AI H9N2 continues to circulate and is frequently linked to secondary bacterial infections such as *Mycoplasma gallisepticum* and *Escherichia coli*, leading to complex respiratory syndromes [5]. Co-infection with multiple pathogens can alter disease dynamics, increase severity, and complicate diagnosis and control measures [6]. Despite their practical importance, field cases involving concurrent viral, bacterial, parasitic, and environmental stressors remain underreported.

In a commercial broiler farm located in Majalengka, West Java, a significant increase in mortality was observed during the grow-out phase, with 419 deaths recorded by 28 days of age from a population of 11,000 birds. The mortality pattern, occurring after the brooding period, combined with the presence of respiratory signs and systemic lesions, strongly suggested involvement of Avian Influenza, especially considering the endemic circulation of AI H5 and H9 subtypes in Indonesia. Such mortality patterns are frequently associated with AI infections complicated by secondary bacterial infections and environmental stressors, which may exacerbate disease severity and obscure the primary cause. This situation underscored the necessity for a comprehensive diagnostic investigation to determine the role of AI and its interaction with other contributing factors.

## 2. Materials and Methods

### 2.1. Case Description and Farm Background

This study describes a field case that occurred in a commercial broiler farm located in Majalengka, West Java, Indonesia, housing approximately 11,000 broiler chickens raised under a closed-house system. The house measured 110 m × 10 m × 2 m and was equipped with six exhaust fans, four of which operated during nighttime ventilation. The flock followed a routine vaccination program starting on day 0, including ND (Vectormune^®^; Ceva Santé Animale, Libourne, France), IBD (Transmune^®^; Ceva Santé Animale, Libourne, France), and a combined ND and IB vaccine (Cevac Vitabron^®^; Ceva Santé Animale, Libourne, France). At seven days of age, the birds were revaccinated with a live ND vaccine (LaSota strain; Medivac^®^ Medion Farma Jaya, Bandung, Indonesia) and an IB vaccine (Ibird^®^; Ceva Santé Animale, France), in accordance with the farm protocol.

During the first farm visit on day 15, birds exhibited lethargy and soft feces. Rectal temperature measurements revealed several individuals with elevated body temperatures ranging from 41.5–41.8 °C. At the second visit on day 21, respiratory signs became more apparent, characterized by audible respiratory sounds (“clicking” or tracheal rales), while soft feces persisted. During the brooding phase on days 0–10, daily depletion remained low and stable (0.05–0.12%), consistent with normal production standards. A slight increase was observed between days 11–14 but remained below the 0.2% daily threshold considered acceptable. A more pronounced increase occurred between days 15–18, when depletion reached 0.23–0.3%, exceeding the 0.2% benchmark with the highest weekly mortality peak recorded during the third week. After day 19, depletion gradually declined to approximately 0.1%, with minor fluctuations (0.08–0.18%) between days 21–25. Diagnostic investigations were conducted at 15, 21, and 28 days of age in response to mortality rates exceeding expected production thresholds.

Supportive management measures were implemented during the outbreak. Probiotic supplementation (Lactoped^®^ Tekad Mandiri Citra, Bandung, Indonesia) was administered from days 15 to 20. Metaphylactic antibiotic treatment consisting of an enrofloxacin–tylosin (Rofotyl^®^ Medion Farma Jaya, Bandung, Indonesia) combination had been given during the first week of age according to farm protocol. Acidifier supplementation (Escent L^®^ Sehat Cerah Indonesia, Subang, Indonesia) was maintained throughout the production cycle. Following these interventions and environmental adjustments, clinical signs gradually decreased, and mortality rates stabilized toward market age.

### 2.2. Pathological Examination

Pathological anatomy examination was performed through necropsy on six chickens exhibiting similar clinical signs. Gross examination included assessment of body condition, external lesions, and visceral organs. After standard necropsy procedures, respiratory organs, digestive tract, liver, heart, kidneys, and lymphoid organs (thymus and bursa of fabricius) were examined macroscopically to identify pathological changes.

### 2.3. Fecal Examination

Parasitological examination was conducted using a fecal flotation technique to detect Eimeria oocysts. Approximately 2 g of feces were mixed with 20 mL of saturated NaCl solution (1:10), homogenized, filtered, and examined using a McMaster counting chamber under light microscopy. Oocyst counts were expressed as oocysts per gram (OPG) of feces.

### 2.4. Bacterial Examination

Bacteriological examination was conducted on yolk sac swab samples collected from necropsied birds. Swabs were inoculated onto MacConkey agar and incubated aerobically at 37 °C for 24 h. Bacterial growth was assessed qualitatively based on colony morphology and lactose fermentation characteristics. The presence of pink colonies was interpreted as indicative of lactose-fermenting Gram-negative bacteria.

### 2.5. Serological Examination

Serological testing was performed on 16 randomly selected serum samples collected at 15, 21, and 28 days of age. This sample size was determined based on routine field diagnostic practices in commercial broiler production, where representative sampling is commonly used for flock-level serological assessment. Hemagglutination inhibition (HI) tests were conducted to detect antibodies against AI subtypes H5 and H9. Infectious Bronchitis antibodies were measured using a commercial ELISA kit (IDEXX^®^ ELISA Kit, Westbrook, ME, USA) following the manufacturer’s instructions. Rapid serum agglutination tests were used to detect antibodies against MG.

### 2.6. Molecular Detection

PCR (Vazyme^®^ Nanjing Vazyme Biotech, Nanjing, China) was performed on organ samples collected from six clinically affected birds that were necropsied during the outbreak. The sampling strategy was targeted rather than random, focusing on birds showing clinical signs to increase diagnostic sensitivity. Tissue collection was based on known pathogen predilection sites. Bursa of Fabricius and cecal tonsils were collected for Infectious Bursal Disease (IBD) detection at 15 days of age. Trachea and lung samples were collected at 21 days of age for detection of Avian Influenza (AI) and Infectious Bronchitis Virus (IBV), guided by gross pathological findings. PCR products were visualized using agarose gel electrophoresis to determine the presence or absence of target viral genomes.

### 2.7. Environmental Assessment

Environmental parameters were evaluated during each farm visit at 15, 21, and 28 days of age. Ambient temperature and relative humidity were measured using a portable digital thermohygrometer (Kestrel^®^; Nielsen-Kellerman Company, Boothwyn, PA, USA). Heat Stress Index (HSI) values were calculated by integrating ambient temperature and relative humidity to estimate the effective thermal load experienced by the birds. Relative humidity values exceeding 80% were considered above the optimal range for broiler production. In this study, the HSI reached values ≥ 160, exceeding the recommended tolerance thresholds for broiler chickens, indicating heat stress conditions. Ammonia concentration was assessed using ammonia indicator paper (Hydrion^®^; Micro Essential Laboratory, Broklyn, NY, USA) based on colorimetric change and expressed in parts per million (ppm).

## 3. Results

### 3.1. Pathological Findings

**Figure 1:**
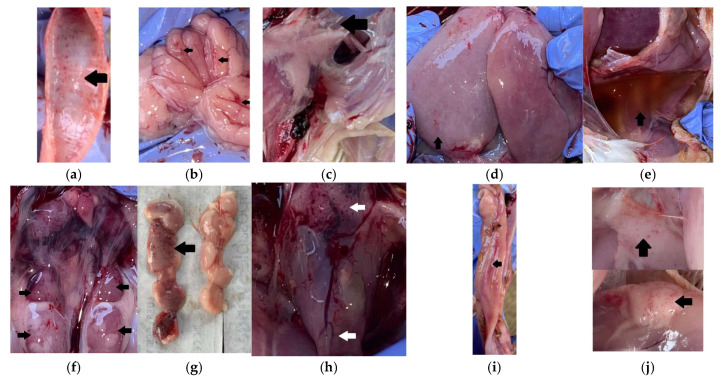
Anatomic pathology findings include: (**a**) Tracheitis; (**b**) Bursitis; (**c**) Airsacculitis; (**d**) Perihepatitis; (**e**) Ascites; (**f**) Swollen kidneys; (**g**) Thymitis; (**h**) Petechiae hemorrhage in the pericardium; (**i**) Enteritis; (**j**). Petechiae hemorrhages in muscle and fat.The black arrow and white arrow show lesion (no different between both color).

Post-mortem examination revealed lesions affecting multiple organ systems, including tracheitis, airsacculitis, thymitis, bursitis, perihepatitis, ascites, nephromegaly, petechial hemorrhages in muscle, fat, and pericardium, as well as enteritis (Figure 1). These findings indicated the involvement of respiratory, lymphoid, circulatory, renal, and gastrointestinal systems.

### 3.2. Fecal Examination

Fecal flotation revealed the presence of Eimeria oocysts with a count of 4400 OPG, corresponding to a mild degree of coccidiosis infection.

### 3.3. Bacterial Examination

Bacteriological examination of yolk sac swab samples from necropsied chickens revealed bacterial growth on MacConkey agar after 24 h of aerobic incubation at 37 °C. Pink colonies were observed, indicating the presence of lactose-fermenting Gram-negative bacteria.

### 3.4. Serological Findings

#### 3.4.1. Hemagglutination Inhibition (HI)

HI testing showed that antibodies against AI H9 at 21 days of age exhibited a post-infection recovery pattern, characterized by a low coefficient of variation (CV < 30%) indicating relatively uniform field exposure within the flock. In contrast, antibodies against AI H5 clade 2.3.2 displayed a highly heterogeneous distribution at 21 days (CV 163.3%), suggesting an early stage of infection. By 28 days of age, the CV decreased to 28.7%, indicating a transition toward a more uniform post-infection antibody distribution (Table 1).

#### 3.4.2. ELISA for Infectious Bronchitis

ELISA results at 28 days showed a high mean antibody titer (854) with a very high CV (256%), indicating a non-uniform antibody distribution. Most samples exhibited low or negative titers, while one sample showed a markedly high titer (9211), exceeding the suspect threshold (>3000), suggesting limited field exposure to IB (Table 2).

#### 3.4.3. Rapid Test for Mycoplasma gallisepticum

Rapid agglutination testing detected antibodies against Mycoplasma gallisepticum in 5 out of 16 samples (31.25%), indicating partial exposure within the flock.

### 3.5. Molecular Detection

PCR assays for AI, IB, and IBD performed on selected organs at 15 and 21 days of age yielded negative results for all targets (Table 3).

### 3.6. Environmental Conditions

**Figure 2:**
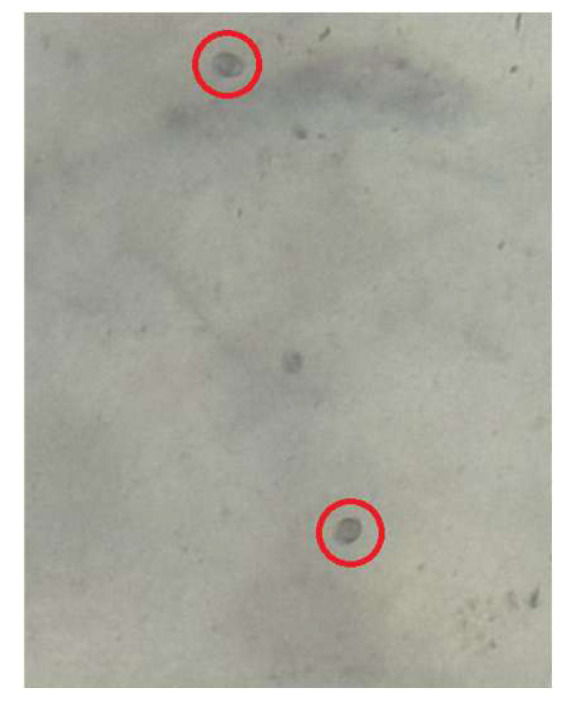
*Eimeria* spp. oocysts observed in fecal samples by flotation technique under light microscopy (400×).

**Figure 3:**
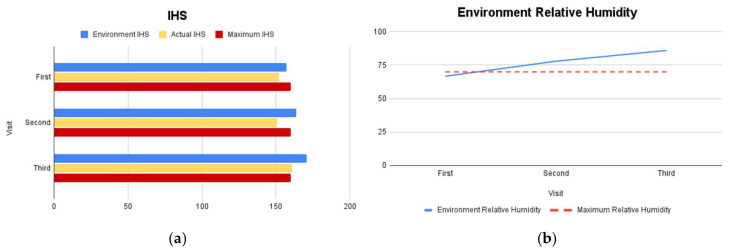
Results of environmental inspection of the cage: IHS (**a**) and relative humidity (**b**).

Environmental monitoring showed ammonia concentrations reaching 20 ppm, relative humidity consistently exceeding 80%, and Heat Stress Index (HSI) values above 160 across all three visits (Figure 2 and Figure 3).

## 4. Discussion

### 4.1. Discussion

The present study demonstrates that disease occurrence in the investigated broiler flock was driven by a complex interaction between multiple infectious agents and unfavorable environmental conditions. Gross pathological examination revealed multisystemic involvement affecting the respiratory, lymphoid, circulatory, renal, and gastrointestinal systems. Respiratory lesions characterized by tracheitis and airsacculitis indicate chronic respiratory damage consistent with complex CRD [6]. These lesions are commonly associated with synergistic interactions between MG and low pathogenic AI subtypes such as H9, which share tropism for the respiratory epithelium and disrupt the mucociliary defense system [7]. Damage to respiratory barriers may have facilitated secondary bacterial invasion, as suggested by the presence of perihepatitis and ascites lesions commonly associated with colibacillosis [3]. The detection of multifocal petechial hemorrhages in skeletal muscle, adipose tissue, and pericardium suggests systemic vascular involvement. Such lesions may be compatible with systemic viral involvement, as certain avian influenza viruses are known to induce endothelial damage and vasculitis, resulting in hemorrhagic manifestations across multiple tissues [4]. In addition, lymphoid lesions including thymitis and bursitis indicate impairment of primary immune organs, reflecting virus-induced immunosuppression [8]. AI infections have been widely reported suppressing T-cell function, macrophage activity, and antibody production, thereby increasing susceptibility to secondary bacterial and parasitic infections [8,9]. However, in the absence of positive molecular detection or histopathological confirmation, these findings cannot be conclusively attributed to active AI H5 infection at the time of sampling and should therefore be interpreted cautiously. Renal enlargement observed during necropsy may be associated with nephropathogenic strains of IB, which are capable of infecting renal tubular epithelium and disrupting fluid and electrolyte balance [10]. Renal involvement can further exacerbate systemic stress and contribute to reduced performance and increased mortality [11]. Gastrointestinal lesions in the form of enteritis, combined with mild coccidial infection detected through fecal examination, suggest that although parasitic involvement was not the primary cause of mortality, it likely acted as a comorbid factor by impairing nutrient absorption and compromising gut-associated lymphoid tissue function [12].

The serological patterns observed in this study suggest distinct infection dynamics between AI H9 and AI H5 within the flock. The relatively low coefficient of variation (CV < 30%) of H9 antibody titers suggests a more uniform exposure among birds, which is consistent with the endemic and often subclinical circulation of low-pathogenic AI H9 in commercial broiler production [13]. Such homogeneous seroconversion patterns have frequently been associated with gradual field exposure rather than acute outbreak events, particularly under conditions of concurrent respiratory stress [13,14]. In contrast, the markedly heterogeneous antibody response against AI H5, as reflected by the extremely high CV values during the early phase of infection, indicates an uneven spread of the virus within the flock [15]. This pattern is characteristic of the early stage of field infection, where only a subset of birds has undergone seroconversion while others remain immunologically naïve [15,16]. The subsequent reduction in variability observed at later sampling points suggests progressive virus dissemination and a transition toward a post-infection stage at the population level. Importantly, the absence of vaccination against both AI H5 and H9 indicates that the detected antibody responses originated from field exposure, as non-vaccinated broilers at approximately three weeks of age typically exhibit negligible AI antibody titers, and elevated titers at this age are therefore strongly suggestive of recent infection [17]. The serological profile of IB at 28 days of age showed an extremely high coefficient of variation (CV = 256%), indicating a highly heterogeneous antibody distribution within the flock [13]. In commercial broiler production, a CV below 40% for inactivated vaccines and below 60% for live vaccines is generally considered indicative of uniform immunization, whereas elevated CV values are strongly associated with uneven immune responses caused by field virus exposure rather than vaccine-induced immunity [18]. In the present case, most birds exhibited low to negative IB antibody titers, while a single individual showed an exceptionally high titer exceeding the suspect threshold, suggesting limited and sporadic exposure to IBV rather than flock-wide infection [19]. Similar serological patterns have been reported in flocks experiencing subclinical Infectious Bronchitis Virus (IBV) circulation, where only a subset of birds is exposed due to compromised mucosal immunity or concurrent respiratory stressors [19,20]. Partial seropositivity to MG further supports the presence of a multifactorial respiratory disease complex. Detection of MG antibodies in approximately one-third of the samples indicates that exposure was not uniform throughout the flock. Because rapid MG tests primarily detect IgM antibodies, positive results are consistent with early or ongoing infection [21]. However, under suboptimal environmental conditions or in the presence of concurrent viral respiratory infections such as AI and IB, MG infections may persist in a chronic or subclinical form, resulting in incomplete seroconversion at the flock level [22,23]. The coexistence of MG with viral respiratory pathogens is well recognized to exacerbate respiratory lesions, impair mucociliary clearance, and facilitate secondary bacterial colonization [24]. Molecular assays for AI, IB, and IBD yielded negative results despite serological and pathological evidence of infection. This discrepancy can be explained by the temporal dynamics of viral replication. PCR detection depends on the presence of viral genetic material during active replication, whereas serological responses persist long after viral clearance [25]. Several studies have demonstrated that PCR sensitivity decreases significantly when sampling is conducted after the peak replication phase, leading to false-negative results in resolving infections [26]. The negative PCR results suggest that viral shedding may have declined or resolved by the time diagnostic samples were collected. Although serological testing demonstrated antibody responses against AI H5 and H9, these findings indicate prior field exposure rather than definitive evidence of active viral replication during sampling.

The environmental conditions identified in this study likely played a crucial role in exacerbating disease severity. Ammonia concentrations reaching 20 ppm are known to damage respiratory epithelium, impair ciliary function, and reduce local immune defenses, thereby increasing susceptibility to airborne pathogens and secondary infections [27]. Additionally, consistently elevated HSI values and excessive humidity indicate chronic thermal stress, which has been associated with immunosuppression, reduced feed intake, and impaired vaccine responsiveness [28]. Excessive humidity further degrades litter quality, facilitating the survival and transmission of pathogens [28]. Taken together, the findings of this case study support a multifactorial disease model in which respiratory viral infections, bacterial pathogens, mild parasitic involvement, and adverse environmental conditions interact to produce severe clinical outcomes. This case highlights that disease control in commercial broiler operations cannot rely solely on vaccination programs but must be integrated with strict environmental management and biosecurity measures. Future research should focus on longitudinal monitoring of pathogen dynamics in broiler flocks, particularly the temporal relationship between serological responses, molecular detection, pathological lesions, and environmental stress indicators. Controlled studies evaluating the combined effects of ammonia exposure, heat stress, and co-infection on immune competence and vaccine performance would provide valuable insights for improving disease prevention strategies in intensive poultry production systems.

When interpreted within a multifactorial framework, the pathological findings in this case suggest a sequential interaction between respiratory viral exposure, opportunistic bacterial colonization, organ-specific complications, and environmental stressors. Serological evidence of prior exposure to avian influenza viruses, together with partial seropositivity to MG, may have contributed to respiratory epithelial damage and impaired mucociliary clearance. Disruption of this primary defense barrier could have facilitated secondary colonization by opportunistic Gram-negative bacteria, as suggested by lesions such as airsacculitis, perihepatitis, and ascites that are commonly associated with colibacillosis. Sporadic serological evidence of IBV exposure, combined with renal enlargement observed during necropsy, raises the possibility of renal tubular involvement. Although molecular assays were negative at the time of sampling, the integration of serological patterns and gross pathological findings supports the interpretation of prior viral exposure rather than confirmed active infection. These infectious processes occurred concurrently with elevated ammonia levels, excessive humidity, and increased HSI values during the third week of age. Such environmental stressors are known to impair respiratory defenses and immune competence, potentially amplifying disease severity and contributing to the observed mortality peak.

### 4.2. Study Limitations

This case report has several limitations that should be acknowledged. First, the investigation was conducted in a single commercial broiler flock, which limits the generalizability of the findings to other production systems. Second, viral isolation and histopathological examination were not performed; therefore, definitive confirmation of active viral infection at the time of sampling was not possible. Third, molecular assays yielded negative results, which may reflect sampling conducted outside the peak phase of viral replication. Consequently, interpretations regarding viral involvement are primarily based on serological patterns, gross pathological findings, and epidemiological context. Despite these limitations, the integration of clinical observations, serology, pathology, bacteriological findings, and environmental assessment provides a comprehensive field-based evaluation of a multifactorial disease event.

## 5. Conclusions

This case report demonstrates that increased mortality in the investigated broiler flock was not attributable to a single etiological agent but was associated with a multifactorial disease process involving prior exposure to avian influenza viruses, secondary bacterial and parasitic involvement, and unfavorable environmental conditions. Serological findings indicated distinct exposure patterns to AI H9 and AI H5 within the flock. However, negative PCR results suggest that active viral replication may not have been present at the time of sampling and therefore the findings are interpreted as evidence of previous field exposure rather than confirmed active co-infection. Gross pathological findings supported the presence of a complex disease interaction affecting multiple organ systems, consistent with respiratory viral involvement complicated by immunosuppression and secondary infections. Environmental stressors including elevated ammonia levels, excessive humidity, and sustained heat stress likely acted as amplifying factors by impairing respiratory defenses and immune competence. Overall, this case underscores that effective disease control in commercial broiler production requires an integrated approach combining vaccination, environmental management, continuous health monitoring, and early detection of multifactorial disease interactions.

## Figures and Tables

**Table 1:** Comparison of HI Titer on Day 21 and Day 28.

	Titer	0	1	2	3	4	5	6	7	8	AVG ^1^	STD ^2^	CV ^3^ (%)
AI H5 2.3.2	21 days	11	2	3	0	0	0	0	0	0	0.5	46.83	163.3
	28 days	0	0	7	5	4	0	0	0	0	2.81	9.56	28.72
AI H9	21 days	0	0	1	5	10	0	0	0	0	3.56	3.98	17.66
	28 days	0	0	0	7	6	2	1	0	0	3.81	7.73	23.88

^1^ AVG: average. ^2^ STD: standard deviation. ^3^ CV: coefficient of variation.

**Table 2:** Comparison of ELISA IB Day 21 and Day 28.

Age	Mean Titer	Std. Mean Titer	Titer Min.	Titer Max	CV (%)
21 days	81	67	3	263	82.9
28 days	854	2188	40	9211	256.4

**Table 3:** Interpretation of PCR test results on Day 15 and Day 21.

Antigens Tested	Age	
	15 Days	21 Days
IBD	-	Not performed
IB	-	-
AI	Not performed	-

## Data Availability

The data presented in this study are available from the corresponding author upon reasonable request. The data are not publicly available due to confidentiality and proprietary restrictions related to the commercial poultry farm and diagnostic records.

## Abbreviations

The following abbreviations are used in this manuscript:

AI	Avian Influenza
IB	Infectious Bronchitis
IBV	Infectious Bronchitis Virus
IBD	Infectious Bursal Disease
CRD	Chronic Respiratory Disease
MG	Mycoplasma gallisepticum
HI	Hemagglutination Inhibition
HSI	Heat Stress Index
PCR	Polymerase Chain Reaction
ELISA	Enzyme-linked Immunosorbent Assay
CV	Coefficient of Variation
OPG	Oocysts Per Gram
